# From stabilization to silencing in transthyretin amyloid cardiomyopathy: why sequencing should be treat-to-trajectory, not ‘one-and-done’

**DOI:** 10.1093/ehjcvp/pvag013

**Published:** 2026-04-28

**Authors:** Arif Albulushi, Tasneem Al Rashdi, Hatem Al-Farhan

**Affiliations:** Department of Adult Cardiology, National Heart Center, The Royal Hospital, PO Box 1331, Muscat 111, Oman; Department of Adult Cardiology, National Heart Center, The Royal Hospital, PO Box 1331, Muscat 111, Oman; Department of Internal Medicine, Sultan Qaboos University Hospital, PO Box 38, Muscat 123, Oman

## Abstract

Transthyretin amyloid cardiomyopathy (ATTR-CM) is now a treatable cause of heart failure, but the rapid expansion of disease-modifying therapies has introduced a practical challenge: how to choose and sequence treatments in routine care. Transthyretin (TTR) stabilizers reduce tetramer dissociation, whereas silencers reduce hepatic TTR production. Although recent randomized trials have strengthened the evidence base, real-world management remains heterogeneous and is often driven by availability rather than phenotype, disease stage, and tempo of progression. We propose a pragmatic treatment framework based on two serial decisions: baseline stage (amyloid burden and organ reserve) and short-interval trajectory assessment using standardized clinical, biomarker, and imaging signals. A stabilizer-first approach is often reasonable in earlier-stage patients with stable trajectories, whereas silencer first or early escalation may be favored when progression is rapid, extracardiac involvement is prominent, or trajectory worsens despite treatment. Combination therapy is biologically attractive but remains an evidence gap in the absence of dedicated randomized sequencing data. This review synthesizes contemporary evidence and translates it into a clinic-ready, treat-to-trajectory approach designed to support reproducible decision-making across amyloid and heart failure programs.

## Graphical Abstract

**Graphical Abstract:**
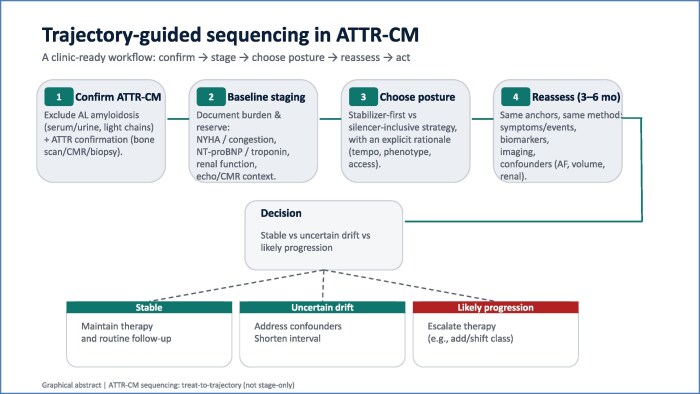
Trajectory-guided sequencing in ATTR-CM: confirm diagnosis (exclude AL), assign baseline stage, choose initial therapy posture (stabilizer-first vs. silencer-inclusive), reassess at 3–6 months using symptoms/events plus biomarkers and imaging, then act—continue if stable, address confounders/shorten follow-up if uncertain, or escalate therapy if progression is confirmed.

Transthyretin amyloid cardiomyopathy (ATTR-CM) is now a common, treatable cause of heart failure. Contemporary guidance has improved diagnostic confidence and encouraged earlier confirmation using structured algorithms, thereby shifting the central clinical question from ‘is this amyloid?’ to ‘how should we treat, monitor, and escalate?’.^1–4^ Yet, real-world care still often follows an implicit, availability-driven default: start a stabilizer and watch.

That default made sense when tafamidis was the only proven disease-modifying option. ATTR-ACT established survival benefit and fewer cardiovascular hospitalizations, and tafamidis remains a foundational therapy.^5^ However, we have moved into a therapeutic plurality. Acoramidis broadened the stabilizer class with outcome-relevant benefit.^6^ More importantly, the cardiomyopathy outcomes era for silencers has arrived: patisiran improved functional capacity and quality of life over 12 months,^7^ and vutrisiran reduced mortality and cardiovascular events while preserving function and quality of life in ATTR-CM.^8^ With these data, the field can no longer treat ‘stabilizer-first for everyone’ as an evidence-neutral stance.

The controversy is not whether stabilization works—it does. The controversy is whether a one-size-fits-all stabilizer-first posture remains defensible when upstream TTR reduction has now demonstrated cardiomyopathy outcomes. The practical challenge is that ATTR-CM progression is heterogeneous, and baseline stage does not reliably predict near-term tempo. Biomarker staging systems (e.g. NAC) are clinically useful for risk conversations,^9^ but natural history cohorts show wide variability in trajectory, symptoms, and event burden even among patients with similar baseline markers.^10^ This heterogeneity is amplified by confounders that can mimic progression: atrial fibrillation, congestion shifts, and renal fluctuation can all distort symptoms and biomarkers if not accounted for.^11^

We argue for a treat-to-trajectory sequencing mindset. This is not a new score. It is a clinic discipline that makes ‘response’ explicit, time-bound, and actionable: (i) confirm diagnosis using standardized pathways and exclude AL amyloidosis^1–4^; (ii) assign baseline stage using organ reserve and biomarkers^9^; (iii) choose an initial posture (stabilizer-first vs. silencer-inclusive) with a stated rationale; (iv) reassess at a short interval (typically 3–6 months) using the same anchors; and (v) escalate only when concordant progression is confirmed and confounders have been addressed.

In practice, trajectory can be communicated with a simple traffic-light language that is easily shared across amyloid and heart failure teams: stable (no consistent worsening across domains), uncertain drift (a single worsening signal, plausibly confounded), or likely progression (concordant worsening, e.g. rising NT-proBNP with worsening congestion burden and functional decline).

A stabilizer-first approach remains appropriate for many patients diagnosed early, with preserved reserve and a stable early trajectory—particularly where access, tolerability, and pill burden favour this option.^5,6^ However, we believe silencer-inclusive strategies should be considered earlier in three common scenarios.

First, rapid tempo. Patients with repeated decompensations, rising biomarker trajectory, or functional decline over a short interval (despite optimized supportive care) should not have to ‘prove’ progression over a year before escalation is considered. Here, upstream TTR reduction offers a mechanistically direct approach, now supported by cardiomyopathy outcomes data.^8^

Second, mixed phenotype and extracardiac involvement. Variant ATTR with neuropathy or autonomic dysfunction is often a systemic disease-control problem rather than an isolated cardiomyopathy problem; a TTR-lowering posture is conceptually aligned with systemic substrate reduction.^12^

Third, late presentation with limited reserve. In advanced disease, sequencing should be goal-aligned. The question is not simply ‘add therapy,’ but whether escalation is likely to preserve meaningful function or reduce event burden within a realistic time horizon. Some patients will benefit from escalation; others will prioritize symptom control and treatment simplicity. A treat-to-trajectory mindset supports either path by requiring an explicit goal and an explicit reassessment plan.

Combination therapy (stabilizer plus silencer) is biologically attractive, but clinically unresolved. Complementary mechanisms do not guarantee additive clinical benefit, and routine combination is difficult to justify without comparative evidence. For now, combination is best viewed as a selective strategy for expert centres and carefully chosen patients with high-risk or progressive trajectories despite therapy, with predefined goals and early reassessment to avoid reflex escalation without measurable rationale.

Supportive cardiovascular care remains the silent determinant of outcomes. Disease-modifying therapy does not replace congestion strategy, rhythm management, anticoagulation, and renal-aware diuretic practice. A trajectory-first model only works if it starts by excluding pseudo-progression driven by atrial arrhythmias, intercurrent illness, or renal injury, and if it acknowledges that ‘worsening’ may reflect physiology rather than treatment failure.^11^ Cost and access also remain practical constraints, particularly for tafamidis, and should be part of shared decision-making rather than an unspoken driver of sequencing.^13^

The sequencing era in ATTR-CM is here. The next step is to make sequencing explicit, reproducible, and auditable. Treat-to-trajectory care—anchored in early reassessment and escalation discipline—offers a practical bridge between current evidence and the comparative trials still needed, while remaining adaptable as the pipeline evolves.^14,15^

## Data Availability

No new data were generated or analysed in support of this Viewpoint.
